# Recombinant characterization and pathogenicity of a novel L1C RFLP-1-4-4 variant of porcine reproductive and respiratory syndrome virus in China

**DOI:** 10.1186/s13567-024-01401-y

**Published:** 2024-11-06

**Authors:** Xinyi Huang, Guoqing Liu, Tong Chang, Yongbo Yang, Tao Wang, Dasong Xia, Xinyu Qi, Xulong Zhu, Ziyi Wei, Xiaoxiao Tian, Haiwei Wang, Zhijun Tian, Xuehui Cai, Tongqing An

**Affiliations:** 1 State Key Laboratory for Animal Disease Control and Prevention, Harbin Veterinary Research Institute, Chinese Academy of Agricultural Sciences, Harbin, 150069 China; 2 Heilongjiang Provincial Key Laboratory of Veterinary Immunology, Harbin, 150069 China

**Keywords:** Porcine reproductive and respiratory syndrome virus, L1C-1-4-4 variant, recombination, pathogenicity, cross-protection

## Abstract

Porcine reproductive and respiratory syndrome (PRRS) is one of the most significant diseases affecting the pig industry worldwide and is caused by the PRRS virus (PRRSV), which has complex genetic variation due to frequent mutations, indels, and recombination. The emergence of PRRSV L1C.5 in 2020 in the United States has raised worldwide concerns about PRRSV with the RFLP 1-4-4 pattern and lineage 1C. However, studies on the pathogenic characteristics, epidemiological distribution, and effectiveness of vaccines against PRRSV with L1C and RFLP1-4-4 pattern in China are still insufficient. In this study, a novel recombinant variant of PRRSV with RFLP 1-4-4 and lineage 1C features, different from L1C.5 in the United States, was isolated in China in 2021. In pathogenicity experiments in specific pathogen-free piglets or farm piglets, 60–100% of artificially infected experimental piglets died with high fever and respiratory symptoms. Inflammatory cytokine and chemokine levels were upregulated in infected piglets. A commercially modified live vaccine against highly pathogenic PRRSV did not provide effective protection when the vaccinated piglets were challenged with the novel L1C-1-4-4 variant. Therefore, this strain merits special attention when devising control and vaccine strategies. These findings suggest that extensive joint surveillance is urgently needed and that vaccine strategies should be updated to prevent the disease from spreading further.

**Supplementary Information:**

The online version contains supplementary material available at 10.1186/s13567-024-01401-y.

## Introduction

Porcine reproductive and respiratory syndrome (PRRS) is characterized by reproductive failure in sows and respiratory symptoms in pigs and causes enormous economic losses in the pork industry worldwide, leading to a cost of $560 million per year in the United States [1]. PRRS virus (PRRSV) belongs to the genus *Betaarterivirus*, family *Arteriviridae*, order *Nidovirales*, and is classified into two species: *Betaarterivirus suid 1* (virus name PRRSV-1) and *Betaarterivirus suid 2* (virus name PRRSV-2) [2]. The PRRSV genome is approximately 15.0 kb in length, with 5′ and 3′ untranslated regions and a poly(A) tail. The genome contains at least 11 open reading frames (ORFs), including ORF1a, ORF1b, ORF2a, ORF2b, ORF3, ORF4, ORF5a, and ORF5–7 [3].

PRRSV has high genetic variation owing to nucleotide mutations, indels, and recombination. The ORF5 gene, which encodes the major viral envelope glycoprotein, shows high genetic diversity and is used to classify PRRSV lineages [4]. Two methods are used to classify PRRSV-2 genetic diversity. The first utilizes restriction fragment length polymorphism (RFLP) in the ORF5 gene, which was proposed in 1998 to distinguish the modified live vaccine RespPRRS/Repro (parent strain VR-2332) from wild-type PRRSV [5]. The second method is based on the phylogenetic relationships between ORF5 gene sequences. Systematic analysis has led to the phylogenetic classification of PRRSV-2 strains into lineages 1–11 [6, 7]. To clarify the genetic diversity within lineage 1, the strains have been further categorized into 9 sublineages, L1A–L1F and L1H–L1J. For L1C, approximately 60% of the United States L1C strains were further classified into L1C.1–L1C.5, whereas NADC30-like strains from China have not been classified into the level below sublineage 1C [7, 8].

In 2006, a highly pathogenic PRRSV (HP-PRRSV) variant characterized by a 30 amino acid (aa) deletion in the NSP2 protein emerged in China and caused approximately 30% mortality in infected pigs [9]. HP-PRRSV subsequently became the dominant strain circulating in China and has been monitored in several East Asian countries. In 2008, the PRRSV NADC30 strain, characterized by a 131 aa deletion in the NSP2 protein, emerged in the United States and was imported into China in 2013 [10]. The NADC30-like strain is currently the dominant strain in China. At the end of 2020, PRRSV L1C.5 was identified in Iowa and Minnesota, with nursery piglets exhibiting respiratory symptoms and a mortality rate of 17.5% [11]. Subsequently, the pathogenicity, epidemiological distribution, and origin of L1C.5 have been studied [12–14], which has raised great concerns about PRRSV belonging to L1C with an RFLP 1-4-4 pattern (PRRSV RFLP 1-4-4 L1C). Notably, L1C.5 and PRRSV RFLP 1-4-4 L1C are distinct. The former refers specifically to the strain that caused the outbreak in the United States, whereas the latter broadly denotes a group of PRRSV strains belonging to the L1C branch, and the RFLP pattern is 1-4-4. The presence of PRRSV RFLP 1-4-4 L1C in China and its pathogenicity and vaccine protection efficacy are not well characterized.

In this study, a novel PRRSV L1C-1-4-4 variant, HuN2021, was first isolated from a pig farm that experienced abortion of pregnant sows and death of piglets, despite these sows having been vaccinated with Ingelvac^®^ PRRS MLV before breeding. The genomic features of HuN2021, its pathogenicity in both specific pathogen-free (SPF) and farm piglets, and the protective efficacy of vaccines against it were evaluated.

## Materials and methods

### Sample collection

In 2021, pigs on a farm in Hunan Province experienced abortion of pregnant sows and death of piglets, with a mortality rate of 30%, despite these sows being vaccinated with the PRRS MLV before breeding. The lungs and lymph nodes of the diseased piglets were collected and sent for viral detection.

### Viral detection, isolation, and genomic sequencing

The tissue samples were homogenized, and the supernatants were used for viral detection by reverse transcription polymerase chain reaction (RT-PCR) and Sanger sequencing. The positive samples were used to isolate viruses from primary porcine alveolar macrophages (PAMs), as previously described [15]. The supernatants containing the isolated virus were then identified by indirect immunofluorescence assay (IFA) [16], using a specific monoclonal antibody targeting the PRRSV M protein [17]. HuN2021 was passaged in PAMs, and the third passage was used for genomic sequencing. Viral RNA was extracted using the TIANamp Virus RNA Kit (TIANGEN, Beijing, China). cDNA was subsequently synthesized using M-MLV (TaKaRa, Dalian, China) and used as the template for RT-PCR. Eight primer pairs were used to amplify the entire genome (Additional file 1) [18]. The PCR products were sequenced via the Sanger method, and Lasergene software (version 7.1) was used to assemble the full-length genomic sequences.

### Phylogenetic analysis

All available ORF5 sequences of PRRSV-2 isolated from 2016 to 2021 (n = 2050) worldwide were downloaded from GenBank and analysed with HuN2021 using the MAFFT software (version 6.935b) [19]. A phylogenetic tree was constructed using the maximum likelihood method with PhyML software (version 3.0) [20], and the reliability of the tree was evaluated using the aLRT SH-like method. Lineages and sublineages were classified according to previous descriptions [7, 8].

### Recombination analysis

The recombination pattern of HuN2021 was analysed using the Simplot (version 3.5.1) and RDP (version 4.96) software, as previously described [21]. Specifically, nine PRRSV-2 sequences of different lineages, including NADC30 (L1), XW008 (L2), MD001 (L3), EDRD-1 (L3), VR-2332 (L5), P129 (L6), SP (L7), JXA1 (L8), and MN30100 (L9), along with HuN2021, were aligned using the MAFFT software. Recombination events were detected using seven methods, including RDP, BOOTSCAN, MAXCHI, CHIMAERA, 3SEQ, GENECONV, and SISCAN, with default settings in RDP4 [22]. Simplot was used to identify recombination breakpoint locations based on the following parameters: 200 base pair (bp) window width and 20 bp step size. The recombination breakpoints divided the HuN2021 sequence into four fragments. Fragments from recombination or non-recombination regions were used to construct phylogenetic trees to confirm the recombination events.

### Viral pathogenicity in piglets

The animal experiment was approved by the Ethics Committee of the Harbin Veterinary Research Institute, Chinese Academy of Agricultural Science (approval number: 230531-02-GR). To investigate viral pathogenicity, two experiments were successively performed using SPF piglets or PRRS-negative farm piglets. For the SPF piglet test, ten four-week-old SPF piglets were purchased from the National Science and Technology Infrastructure Center (Harbin, China) and randomly divided into HuN2021-infected (*n* = 5) and uninfected (*n* = 5) groups in two separate BSL-2 rooms. Ten four-week-old PRRSV antigen and antibody-negative farm piglets were purchased from a pig herd in Harbin and randomly divided into HuN2021-infected and uninfected groups (*n* = 5 for each group) in two separate BSL-2 rooms. These piglets were also free from porcine circovirus type 2 (PCV2), pseudorabies virus (PRV), and classical swine fever virus (CSFV). Each piglet in the infected group was inoculated intranasally (2 mL) or intramuscularly (1 mL) with HuN2021 (7.8 × 10^10^ viral RNA copies/mL). Uninfected piglets were inoculated in Roswell Park Memorial Institute (RPMI) 1640 medium. The rectal temperature and clinical symptoms of each piglet were monitored and scored daily. Serum, nasal swabs, and anal swabs were collected at 0, 3, 7, 10, 14, and 21 days post-infection (dpi). All the animals were euthanized at 7 dpi (SPF piglets) or 21 dpi (farm piglets), and the lungs, tonsils, thymus, lymph nodes, spleens, kidneys, livers, duodenums, and hearts were collected for viral load determination and histopathological examination. Body weight and thymus weight were recorded to assess weight loss and the degree of thymus atrophy, respectively. Daily weight gain was calculated by subtracting the pre-challenge weight (kg) from the weight at death (kg) and then dividing by the number of days survived. The level of thymus atrophy was assessed using the thymus-to-body weight ratio, which was calculated by dividing the thymus weight (g) by the body weight (kg) and then averaging the ratios over the survival days. In pathogenicity experiments on farm pigs, HuN2021 and HLJ13 were evaluated together, which was similar to data from the uninfected control group [15].

### In vivo immunoprotection test

Fifteen four-week-old PRRSV antigen and antibody-negative piglets were purchased from a pig herd in Harbin and randomly divided into three groups: nonvaccinated (*n* = 5), HP-PRRS MLV-vaccinated (*n* = 5), and NADC30-like candidate MLV-vaccinated (*n* = 5), which were vaccinated intramuscularly with 1 mL of Dulbecco’s modified Eagle’s medium (DMEM), HP-PRRS MLV, and NADC30-like candidate MLV, respectively (the vaccine information is available upon request). At 28 days post-vaccination (dpv), all piglets were challenged intranasally (2 mL) or intramuscularly (1 mL) with HuN2021 (7.8 × 10^10^ viral RNA copies/mL). Rectal temperature and clinical symptoms were recorded daily. Serum and tissues were collected for viral detection, as described above.

### Viral detection by quantitative RT-PCR (RT-qPCR)

Viral RNA was extracted from 140 μL of serum or 0.2 g of tissue using TIANamp Virus RNA Kit (TIANGEN, Beijing, China). RT-qPCR was performed using primers and probe targeting PRRSV-2 as previously described (Additional file 1) [23]. Tissues and serum samples from all pigs in the animal experiments were tested. For the serum samples, the virus loads at different time points were assessed. In the SPF piglet challenge experiment, the viral loads in the serum at 0, 3, and 7 dpi or at the terminal stage were detected. In the farm piglet challenge experiment, the viral loads in the serum at 0, 3, 7, 10, 14, and 21 dpi or at the terminal stage were detected. In the immunoprotection test, the serum samples were tested at 0, 7, 14, and 21 dpv and at 0, 7, 14, and 21 days post-challenge (dpc).

### Detection of PRRSV antibodies

Serum antibodies against PRRSV were determined by enzyme-linked immunosorbent assays (ELISAs) via the PRRSV antibody kit 2XR (IDEXX, USA) according to the manufacturer’s instruction. A sample-to-positive (S/P) ratio greater than or equal to 0.4 was considered positive. In the farm piglet challenge experiment, serum samples were tested at 0, 7, 10, 14, and 21 dpi. In the immunoprotection test, serum samples were tested at 0, 7, 14, and 21 dpv, as well as at 0, 7, 14, and 21 dpc.

### Cytokine measurement by ELISA or RT-qPCR

Commercial pig cytokine ELISA kits for interleukin-6 (IL-6), IL-1β, IL-10, and tumor necrosis factor-α (TNF-α) (Solarbio, Beijing, China) were used to test the levels of cytokines in the serum at 0, 3, and 7 dpi and in the lung homogenate collected during dissection. The validation of cytokine mRNA levels utilized lung tissues from all five piglets in the SPF pig challenge trial and intestinal tissues from four of five pigs (the intestinal tissue of one pig was not collected). Specific primers were designed with Oligo software (version 6.24) according to the published porcine *IL-1β*, *IL-10*, *TNF-α*, *CCL8*, *MCP-1*, and *IFN-γ* sequences in GenBank (Additional file 2). *IL-6*, *IL-8*, and *GAPDH* primers were used as described previously [24, 25]. A UNlQ-10 Column TRIzol Total RNA Isolation Kit (Sangon, Shanghai, China) was used to extract total RNA from the tissue samples. Total RNA was reverse-transcribed using HiScript III All-in-One RT SuperMix Perfect for qPCR (Vazyme, China). qPCR was then performed using ChamQ SYBR^®^ qPCR Master Mix (Vazyme, China) and the QuantStudio 5 system (Applied Biosystems, USA). The relative expression levels were normalized to those of the control gene *GAPDH* using the 2^−ΔΔCT^ method [26].

### Statistical analysis

All the data are presented as the means ± standard deviations (SD). Statistical differences were determined using *t* tests in GraphPad Prism 6 software (version 6.01). A *p* value less than 0.05 was considered statistically significant.

## Results

### Isolation and identification of novel L1C-1-4-4 PRRSV HuN2021

RT-PCR and sequencing of the ORF5 gene identified positive tissue homogenates that were used for viral isolation by inoculation into PAMs. The mixture of supernatant and cells was subsequently harvested and passaged three times, and RT-PCR was used to detect PRRSV positivity and the negativity for other pathogens, such as CSFV, porcine epidemic diarrhea (PEDV), and PRV. The PRRSV strain was designated PRRSV HuN2021 and was identified by IFA using a monoclonal antibody against the M protein of PRRSV (Additional file 3).

### Genomic characteristics of novel L1C-1-4-4 PRRSV HuN2021

**Figure 1:**
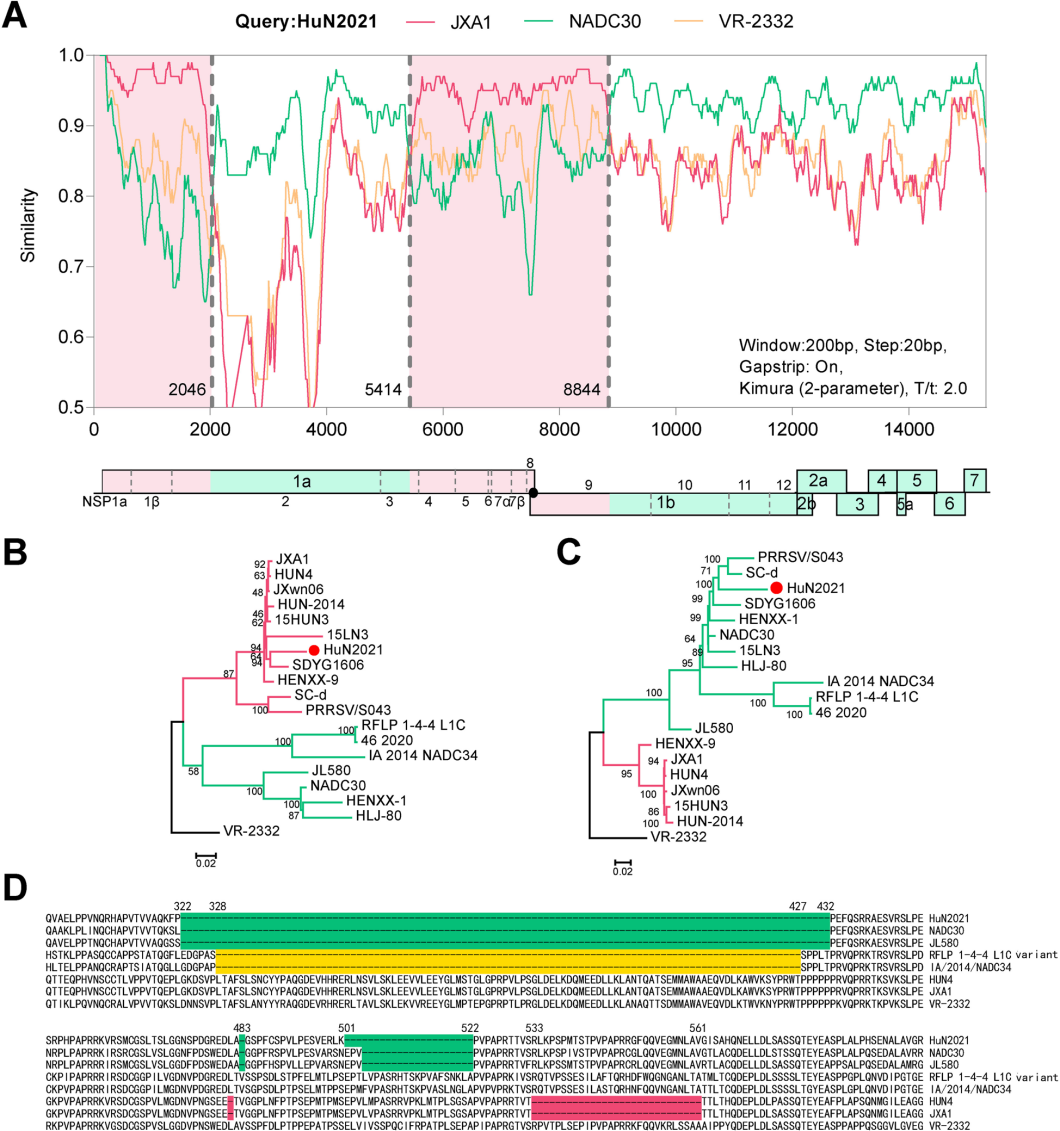
**Recombination analysis of HuN2021.** **A** Recombination detection of HuN2021 using Simplot. **B**, **C** Phylogenetic trees based on the recombinant or non-recombinant regions of HuN2021, respectively. **D** Alignment of HuN2021 with representative strains on the basis of partial NSP2 sequences. The deletions of amino acids are marked by colors, and the positions are labelled on the sequences.

The entire genome of HuN2021 was 15 008 nucleotides (nt), excluding the poly(A) tail at the 3′ end. NSP2 included a 131 aa deletion (111 + 1 + 19), which was the same as that in the NADC30 strain. Additionally, there was an extra deletion of three amino acids at positions 501–503 aa in NSP2 (Figure 1D). Compared with those of representative strains, including VR-2332, QYYZ, CH-1a, JXA1, NADC34, NADC30, and the RFLP-144 L1C variant (L1C.5), most genes of HuN2021 (including NSP10-12, GP2-3, and GP5-N) shared the highest nucleotide identity with the NADC30 strain (91.0–95.7%), whereas NSP1 and NSP4–NSP9 presented the highest similarity with the JXA1 strain (92.7–97.7%) (Additional files 4 and 5). Recombination analysis revealed that HuN2021 is a mosaic virus, with NADC30-like PRRSV and JXA1-like PRRSV providing genomic fragments during its generation. The recombination breakpoints were located at 2046, 5414, and 8844 nt, according to the position in the prototype VR-2332 strain (Figure 1A). On the basis of the recombination breakpoints, the HuN2021 genome was separated into four regions, and two large regions (5414–8844 nt and 8844–15 008 nt) were used to construct the phylogenetic tree. The topological structure of the phylogenetic tree indicated that HuN2021 was a recombinant PRRSV (Figures 1B and C).

### Epidemiologic distribution of RFLP 1-4-4 L1C PRRSV from 2016 to 2021

**Figure 2:**
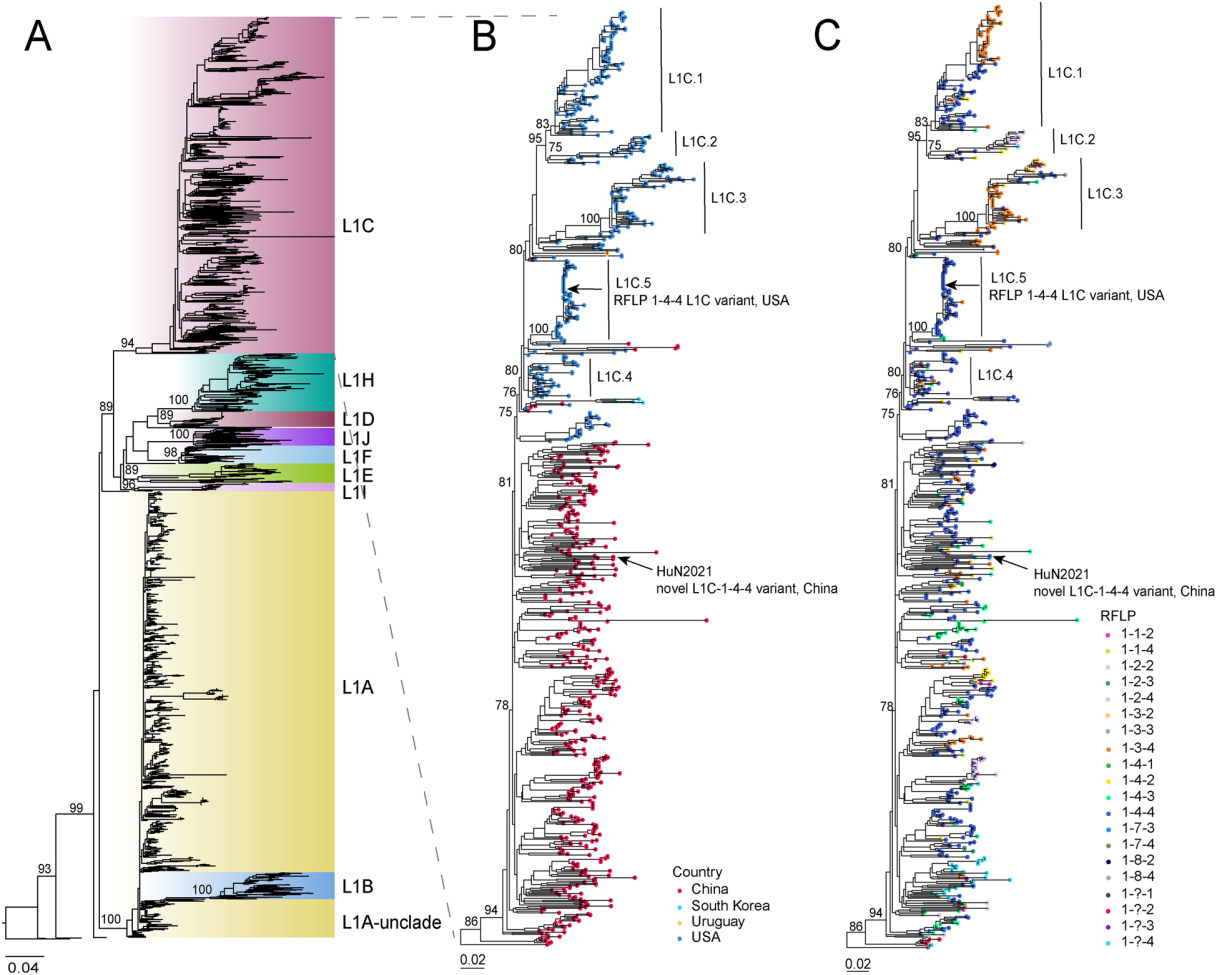
**Genotyping of L1 and L1C PRRSV strains, 2016–2021.** **A** Maximum-likelihood trees of L1 PRRSVs based on ORF5 sequences. The sublineage classification is labelled on the right side of the tree. **B**, **C** Geographical distribution and RFLP patterns of L1C PRRSVs are labelled with solid circles filled with different colors. HuN2021 and L1C.5 are marked with black arrows.

To investigate the epidemiological characteristics of RFLP 1-4-4 L1C PRRSVs, all available L1 PRRSV sequences (*n* = 1785) worldwide in GenBank from 2016 to 2021 and 466 L1 PRRSV reference sequences were analysed. In the phylogenetic tree, the sequences were divided into sublineages L1A–L1F and L1H–L1J (Figure 2A). The L1C PRRSVs were distributed in China (*n* = 455), the United States (*n* = 361), South Korea (*n* = 4), and Uruguay (*n* = 3) (Figure 2B). RFLP analysis of the L1C PRRSVs revealed that HuN2021 was an RFLP 1-4-4 L1C PRRSV, and there were 20 RFLP patterns in L1C PRRSVs. RFLP 1-4-4 was the most dominant pattern (51.8%, 426/823), followed by RFLP 1-3-4 (16.4%, 135/823) and RFLP 1-4-3 (7.0%, 58/823) (Figure 2C). Most L1C PRRSVs from the United States were in RFLP 1-4-4 (*n* = 198) and RFLP 1-3-4 (*n* = 101), whereas Chinese L1C PRRSVs were mostly in RFLP 1-4-4 (*n* = 224) and RFLP 1-4-3 (*n* = 49) (Figure 2C), suggesting that RFLP 1-4-4 is the most common pattern in circulating PRRSV strains in China and the United States. Only four sequences from South Korea belonged to RFLP 1-4-4. The L1C phylogenetic tree revealed that HuN2021 clustered with strains from China, whereas L1C.5 clustered with strains from the United States, which formed a distinct small branch. These findings indicate that although HuN2021 belongs to sublineage 1C and shares the RFLP 1-4-4 pattern, it does not share recent common ancestry with L1C.5.

### Pathogenicity in SPF piglets

**Figure 3:**
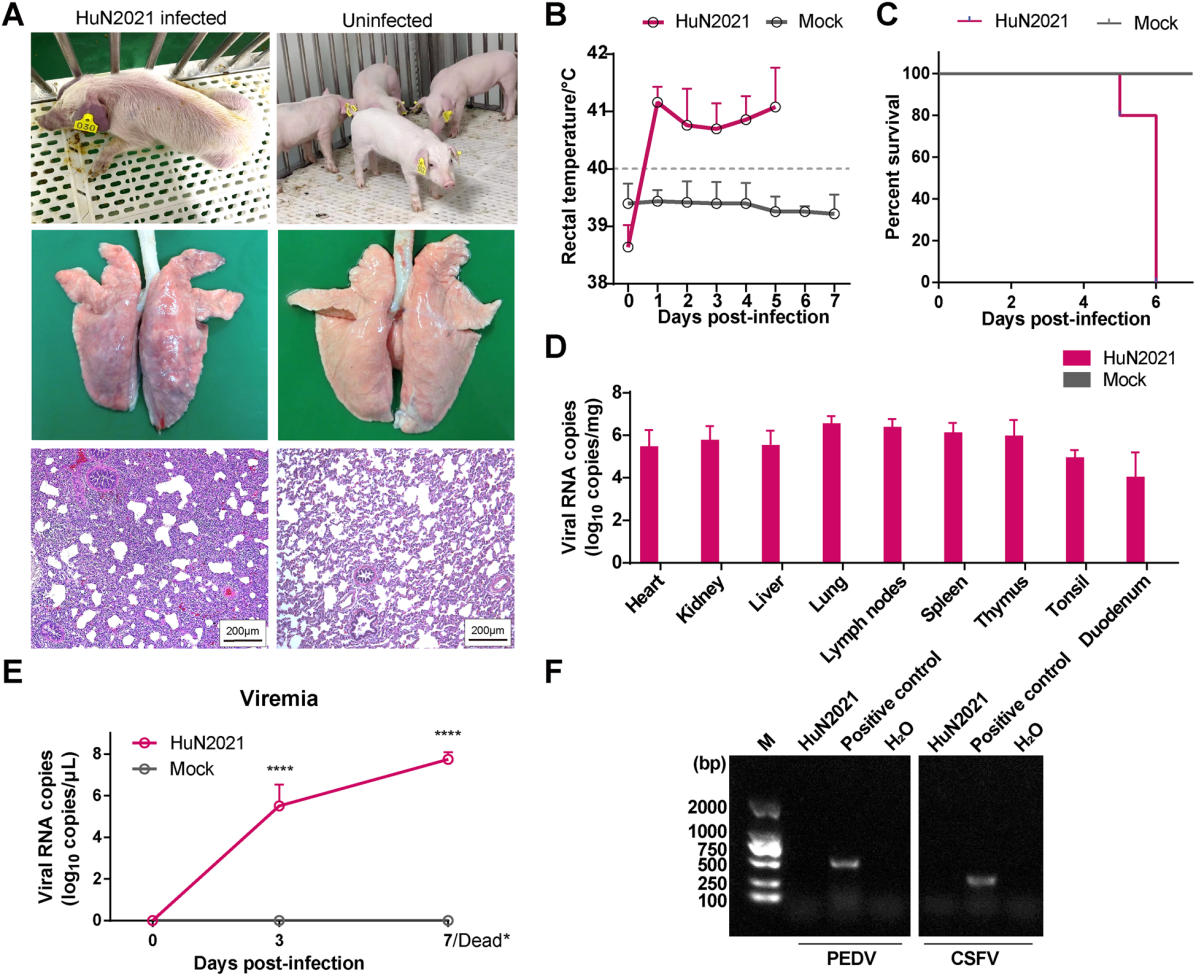
**Pathogenicity evaluation of HuN2021 in SPF piglets.** **A** Clinical signs, gross pathology and microscopic lesions of HuN2021-infected piglets compared with those of uninfected controls. The bar representing the histological images of the lesions represents 200 μm. **B** Mean rectal temperature. The fever cut-off value was set at 40.0 °C. **C** Survival rate. **D** Viral RNA copies in the heart, kidney, liver, lung, lymph nodes, spleen, thymus, tonsil, and duodenum identified by RT-qPCR. **E** Viral loads in serum at 0, 3, and 7 dpi. ****p < 0.0001. Dead* refers to the serum collected from pigs infected with HuN2021 that died at 5 or 6 dpi. **F** RT-PCR was used to determine the absence of PEDV and CSFV in the HuN2021-infected intestine.

The SPF piglets challenged with HuN2021 presented severe clinical signs, including loss of appetite, ear cyanosis, and dystaxia (Figure 3A), and presented persistent fever (> 40.0 °C) throughout the entire experiment (Figure 3B). All the piglets (5/5) died on the sixth day after challenge with HuN2021, whereas all the piglets (5/5) in the uninfected group survived (Figure 3C). Analysis of macroscopic lung lesions revealed that all piglets challenged with HuN2021 presented interstitial pneumonia, characterized by severe tissue consolidation and hemorrhage (Figure 3A). Microscopic examination of lung lesions revealed marked thickening of the alveolar septa and lymphocyte infiltration in HuN2021-inoculated piglets (Figure 3A). In contrast, no obvious clinical signs or pathological lesions were observed in the uninfected group (Figure 3A). RT-qPCR revealed that HuN2021 exhibited extensive tissue tropism, and the viral loads in lung, heart, kidney, liver, lymph node, spleen, thymus, tonsil, and duodenum tissues were significantly different between the HuN2021-infected and uninfected groups (Figure 3D). High-level viremia was observed in HuN2021-infected piglets, with a viral load in the serum of 10^5.5^ copies/μL at 3 dpi, which increased until death (Figure 3E). The viral load in the duodenum was 10^4.0^ copies/mg, which was probably responsible for diarrhea in HuN2021-infected piglets since the intestinal tissue was free of PEDV and CSFV (Figure 3F).

### Pathogenicity in farm piglets

**Figure 4:**
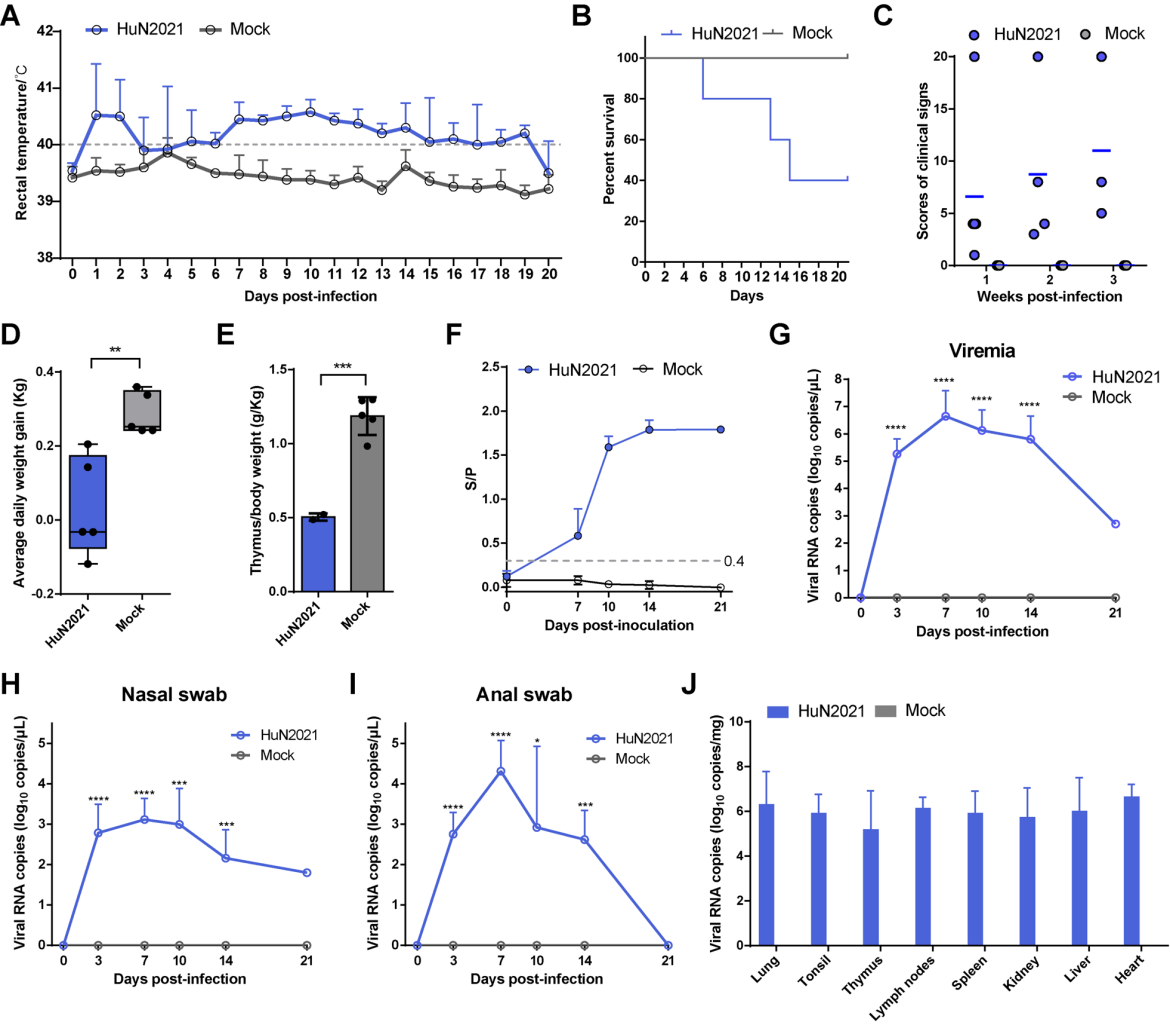
**Pathogenicity evaluation of HuN2021 in farm piglets.** **A** Mean rectal temperature. A temperature exceeding 40.0 °C was considered fever. **B** Survival rate of piglets in the 21-day animal trial. **C** Clinical sign scores at the first, second and third weeks post-infection. **D** Daily weight gain. **E** The level of thymus atrophy. **F** Detection of PRRSV antibodies in the serum at 0, 7, 10, 14 and 21 dpi. S/P ≥ 0.4 was considered positive. The viral loads in the serum (**G**), nasal swabs (**H**), anal swabs (**I**) and tissues (**J**) were detected via RT-qPCR. *p < 0.05; ***p < 0.001; ****p < 0.0001.

Farm piglets infected with HuN2021 experienced two waves of high fever (Figure 4A). The average rectal temperature of infected piglets was approximately 40.5 ℃ at the first 2 dpi, decreased to 40.0 ℃ at 3–6 dpi, and then increased to 40.4–40.6 ℃ at 7–11 dpi before slightly decreasing at 12–20 dpi. Three HuN2021-infected piglets died at 6, 13, and 15 dpi, and only two survived until 21 dpi (Figure 4B). Compared with the uninfected piglets, HuN2021-infected piglets presented obvious clinical symptoms (Figure 4C), especially respiratory and nervous signs. Compared with the uninfected group, the HuN2021-infected group had a significantly lower daily body weight (*p* < 0.01) (Figure 4D) and significant thymus atrophy (*p* < 0.001) (Figure 4E). At 7 dpi, PRRS antibody detection revealed that four of the five HuN2021-infected piglets were positive, and the other piglet died at 6 dpi (Figure 4F). The viral load in the serum of HuN2021-infected piglets was significantly greater than that in the uninfected piglets from 3 dpi (*p* < 0.0001) and reached a peak of 10^6.6^ copies/µL serum at 7 dpi (Figure 4G). The viral load was detectable in the nasal and anal swabs of infected piglets at 3 dpi, peaked at 7 dpi, and persisted up to 14 dpi (Figures 4H, I). Similarly, the virus was detected in a variety of tissues (Figure 4J), suggesting that the HuN2021 strain has broad tissue tropism.

### Upregulation of inflammatory cytokines and chemokines in HuN2021-infected piglets

**Figure 5:**
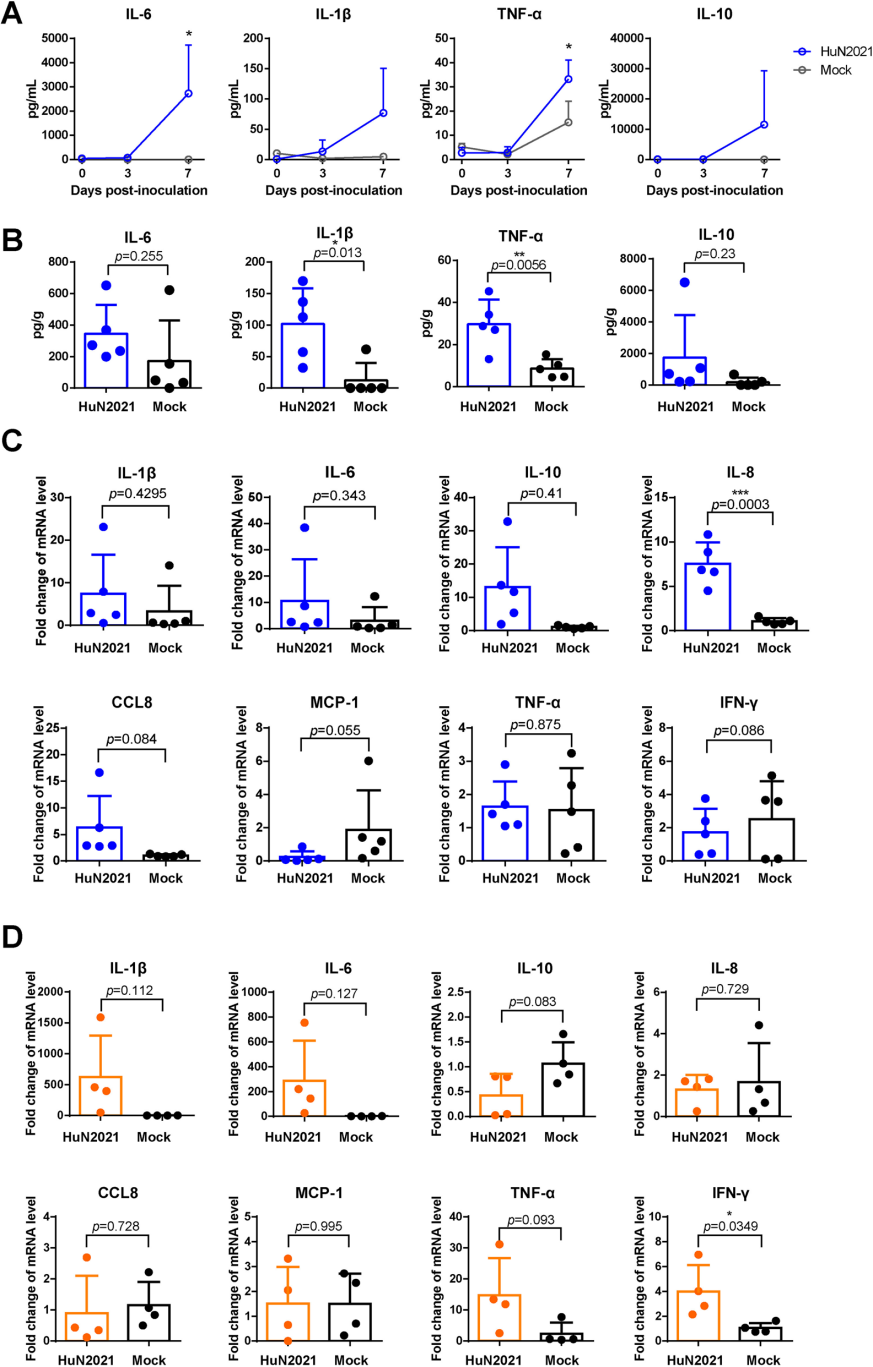
**Cytokine levels in HuN2021-infected piglets.** **A**, **B** Levels of IL-6, IL-1β, TNFα, and IL10 in the serum (**A**) and lungs (**B**) were determined by ELISA. **C**, **D** mRNA levels of *IL-1β*, *IL-6*, *IL-8*, *IL-10*, *TNF-α*, *CCL8*, *MCP-1*, and *IFN-γ* in the lung **C** and intestine (**D**), as determined by RT-qPCR. The relative expression levels were calculated via the 2^−ΔΔCT^ method and normalized to the level of *GAPDH*. All the data are presented as the means ± SD (error bars). *p < 0.05; **p < 0.01; ***p < 0.001.

To investigate whether the high mortality of HuN2021 was related to an excessive inflammatory response, the levels of inflammatory and anti-inflammatory cytokines were measured in serum and lung tissue suspensions by ELISA. Compared with those in the uninfected group, the levels of IL-6, IL-1β, TNF-α, and IL-10 in the serum were increased at 7 dpi, and the levels of IL-6 and TNF-α (*p* < 0.05) were significantly increased (Figure 5A). Similar trends were observed in the lungs of piglets infected with HuN2021, where the levels of IL-6, IL-1β, TNF-α, and IL-10 increased, and the levels of IL-1β and TNF-α significantly differed from those in the uninfected group (Figure 5B). One of the infected piglets (#29) presented significant upregulation of several cytokines in the lungs. mRNA expression analysis revealed significant upregulation of the cytokine IL-8 (*p* < 0.001) in the lungs of HuN2021-infected piglets (Figure 5C). IL-1β, TNF-α, IL-6, and INF-γ were upregulated in the intestinal tissue of HuN2021-infected piglets (Figure 5D).

### Cross-protection of PRRS MLV vaccines against HuN2021

**Figure 6:**
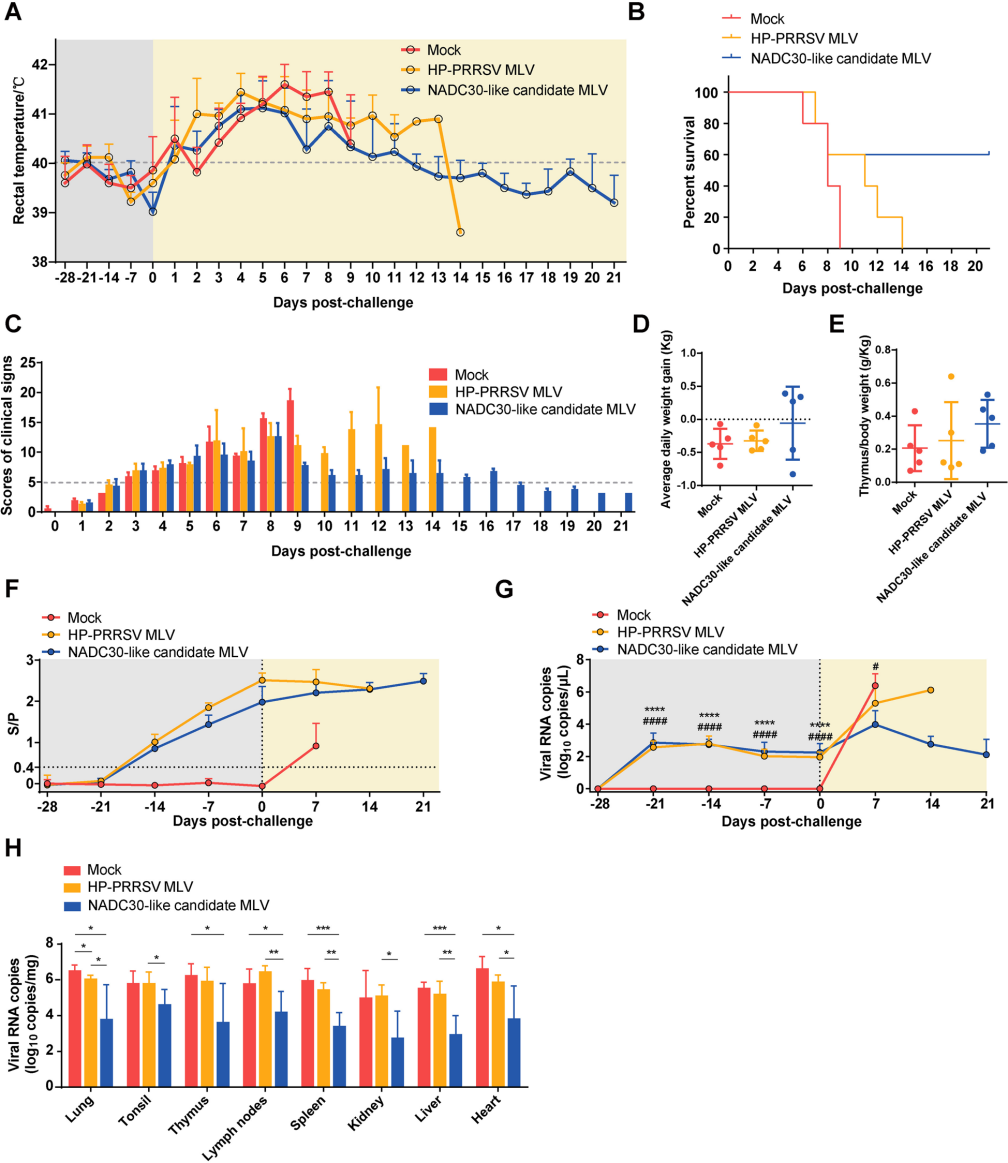
**Protective efficiency of MLVs against HuN2021 challenge.** **A** Rectal temperature. The grey rectangle indicates days post vaccination. The yellow rectangle indicates days post challenge. **B** Survival rate throughout the 21-day experiment. **C** Clinical sign scores. A score of 5 points is indicated by a dashed line. **D** Average daily weight gain. **E** The level of thymus atrophy. **F** PRRSV antibody levels in serum, as determined by ELISA. S/P ≥ 0.4 was regarded as positive. **G** Viral RNA copies in the serum were detected by RT‒qPCR. *indicates a significant difference between the HP-PRRS MLV and Mock group (****p < 0.0001). #indicates a significant difference between the NADC30-like candidate MLV and Mock group (#*p* < 0.05; ####*p* < 0.0001). (H) Viral load in tissues detected by RT-qPCR. *p < 0.05; **p < 0.01; ***p < 0.001.

To evaluate the cross-protection of PRRS MLVs, piglets were immunized with a commercial HP-PRRS MLV, an NADC30-like candidate MLV, or DMEM and then challenged with the HuN2021 strain. All the rectal temperatures were below 40.0 ℃ before the challenge at 28 dpv. In the HP-PRRS MLV- and NADC30-like candidate MLV-vaccinated groups, the temperature rose at 1-4 dpc, peaked at 4 dpc, and then returned to normal gradually. The nonvaccinated group peaked temperature of 41.6 ℃ at 6 dpc (Figure 6A). During the experiment, the nonvaccinated group presented the shortest disease course, and all five piglets died before 9 dpc. The HP-PRRS MLV-vaccinated piglets subsequently died gradually until 14 dpc. The NADC30-like candidate MLV-vaccinated group presented longer survival times, and three of the five piglets survived at 28 dpc (Figure 6B). In both the vaccinated and nonvaccinated groups, clinical signs, such as fever, depression, and coughing, were observed. The clinical sign scores in the nonvaccinated group increased from 1 to 9 dpc; however, in both vaccinated groups, the clinical signs began at 1 dpc and were alleviated by 8 dpc. Compared with the HP-PRRS MLV group, the NADC30-like candidate MLV group presented a more significant reduction in clinical signs (Figure 6C). Following HuN2021 challenge, nonvaccinated and HP-PRRS MLV-vaccinated piglets exhibited rapid weight loss. However, three of the five piglets in the NADC30-like MLV group experienced weight gain, although there was no significant difference between the groups owing to large individual variations within the groups (Figure 6D). Similarly, piglets in both the nonvaccinated and HP-PRRS MLV-vaccinated groups demonstrated more severe thymic atrophy than did those in the NADC30-like candidate MLV-vaccinated group, although this difference was not statistically significant (Figure 6E).

PRRSV-specific antibodies were measured, and the results revealed serum conversion in the two vaccinated groups at 14 dpv (S/P > 0.4). No PRRS antibodies were detected in the nonvaccinated group before challenge. After challenge with HuN2021, the vaccinated groups maintained higher antibody levels than the nonvaccinated groups did (Figure 6F). The viral load in both the serum and tissue was detected by RT‒qPCR, and slight viremia was detected at 7 dpv in both vaccinated groups. After challenge with HuN2021, the viral load in the serum increased in all piglets, and a lower viral load was observed in the two vaccinated groups than in the nonvaccinated group (Figure 6G). The viral load in the lungs of both vaccinated groups was significantly reduced (*p* < 0.05). In particular, the NADC30-like candidate MLV group presented a significant reduction in the viral load in various tissues, including the thymus, lymph nodes, spleen, liver, and heart (Figure 6H).

## Discussion

PRRS is one of the most severe infectious diseases in pigs, resulting in significant economic losses to the pig industry worldwide. There are four lineages of PRRSV-2 in China: NADC30-like (lineage 1), QYYZ-like (lineage 3), VR-2332-like (lineage 5), and HP-PRRSV-like (lineage 8) [27]. Lineages 1 and 8 have been the dominant strains in China since 2015. L1C.5 emerged in the United States at the end of 2020, causing severe abortion in sows and respiratory signs in piglets, which has drawn global attention to this variant [12]. As several RFLP 1-4-4 L1C strains were isolated from 17 provinces in China from 2016 to 2021 (Additional file 6), further research into the genomic characteristics and pathogenicity of the RFLP 1-4-4 L1C strain as well as the protective efficiency of the current MLVs against it is warranted.

HuN2021 is a recombinant strain between NADC30-like PRRSV (lineage 1C) and HP-PRRSV JXA1 (lineage 8E). NSP1, NSP4–NSP8, and partial NSP9 were derived from the JXA1 strain in HuN2021. Because the JXA1 wild-type and JXA1-R MLV strains share high identity, we further confirmed that HuN2021 is a recombinant strain of JXA1 and not JXA1-R because of the absence of amino acids specific to JXA1-R in HuN2021. L1C.5 from the United States was analysed and found to be an intralineage recombinant strain among L1A, L1C, and L1H [13]. Therefore, despite HuN2021 and L1C.5 having the same RFLP 1-4-4 pattern and L1C phylogenetic tree cluster, their viral origins are different. L1 PRRSVs exhibit high-frequency recombination. Recombination increases genetic diversity and favours escape from immune supervision, increasing pathogenicity and causing new waves of outbreaks [18, 28–30].

In this study, a high viral load in the intestine was detected in HuN2021-infected piglets, which matched the clinical signs of diarrhea. Similar gastrointestinal signs are observed in NADC30-like PRRSV-infected piglets [31]. Therefore, HuN2021 provides more evidence that NADC30-like PRRSV gains more tissue tropism in the intestine, although further confirmation is needed.

HuN2021 infection was accompanied by the upregulation of proinflammatory cytokines, such as IL-6, IL-1β, IL-8, TNF-α, and CCL8, as well as the immunomodulatory cytokines IL-10 and IFN-γ, at the mRNA and protein levels. Excessive cytokine secretion is more likely to occur in highly virulent strains. In previous studies, HP-PRRSV BB0907 strain infection increased IL-1β, IL-6, TNF-α, and IFN-γ levels in the serum [32], and infection with the highly pathogenic PRRSV-1 Lena strain increased the serum levels of IFN-γ and IL-6 [33]. Dysregulation of virus-induced cytokines can shift the immune response from anti-inflammatory to proinflammatory, resulting in excessive migration of immune cells, such as neutrophils and macrophages, to the infected region, leading to strong inflammation and tissue damage. Among the prominent cytokines found in PRRSV infection, IL-1β can trigger an inflammatory response [34], IL-6 and TNF-α can activate various immune cells [35, 36], IL-8 is known to recruit and activate neutrophils [37], and CCL8 recruits both monocytes and macrophages [38]. Excessive expression of cytokines may induce a “cytokine storm”, which is correlated with high mortality rates. In patients infected with influenza virus H5N1, high levels of chemokines and cytokines have been observed [39]. Similarly, patients with severe COVID-19 were found to have high plasma levels of IL-10, MCP-1, and TNF-α [40]. Although PRRSV can induce cytokine overproduction [41], the specific mechanisms underlying the excessive secretion of cytokines and the associations among cytokine secretion, the viral load, and disease severity require further investigation.

Coinfection with various pathogens is a common phenomenon in the pig industry. Our previous total infectome approach identified complex interactions between multiple infectious agents, within which coinfections were the norm [42]. Under natural conditions, swine infected with PRRSV often exhibit a high rate of coinfection with various bacteria [42, 43]. Previous studies have shown that HP-PRRSV accelerates *Haemophilus parasuis* infection [44]. Furthermore, in vivo experiments have demonstrated the synergistic effects of PRRSV and *Streptococcus suis* on the morbidity and severity of clinical signs in pigs [45, 46]. In HuN2021-infected farm piglets, thoracic effusion and pulmonary fibrosis were observed during necropsy. 16S rRNA gene PCR results indicated coinfection with *Mycoplasma hyorhinis* or *Pasteurella multocida*. PRRSV-induced inflammation can result in tissue damage and compromise the integrity of the respiratory tract, providing entry points for bacteria to invade and establish infections. Coinfection induces synergistic effects of proinflammatory cytokines [47, 48], which may contribute to the rapid mortality and severity of lung tissue damage observed in HuN2021 infections.

In conclusion, our study revealed that the novel L1C-1-4-4 variant HuN2021 has a mosaic genome and exhibits immunological escape from the currently available HP-PRRS MLV vaccines, which makes prevention via current control strategies difficult. Therefore, monitoring the dynamics of PRRSV with 1-4-4 and L1C characteristics and updating control measures to prevent the further spread of this disease are crucial.

## Supplementary Information

**Additional file 1.** **Primer/probe sequence information for RT-PCR or RT-qPCR assays.**

**Additional file 2.** **Primers used for detecting cytokines by RT-qPCR assay.**

**Additional file 3**. **Isolation of the PRRSV HuN2021 strain.** An indirect immunofluorescence assay was performed with an anti-PRRSV M monoclonal antibody. The bar represents 200 μm.

**Additional file 4. Nucleotide identity of HuN2021 with representative strains.**

**Additional file 5.** **Amino acid identity of HuN2021 with representative strains.**

**Additional file 6.** **Geographical distribution of RFLP 1-4-4 L1C PRRSV strains isolated in China from 2016–2021.** The number of RFLP 1-4-4 L1C PRRSVs in different provinces: Shandong, Henan, Guangdong, Sichuan, Hebei, Fujian, Jiangsu, Heilongjiang, Hunan, Beijing, Guizhou, Jiangxi, Shanxi, Anhui, Yunnan, and Zhejiang.

## Data Availability

The data that support the findings of this study are available from the authors upon reasonable request.
