# Correction: Recombinant characterization and pathogenicity of a novel L1C RFLP-1-4-4 variant of porcine reproductive and respiratory syndrome virus in China

**DOI:** 10.1186/s13567-025-01710-w

**Published:** 2026-01-13

**Authors:** Xinyi Huang, Guoqing Liu, Tong Chang, Yongbo Yang, Tao Wang, Dasong Xia, Xinyu Qi, Xulong Zhu, Ziyi Wei, Xiaoxiao Tian, Haiwei Wang, Zhijun Tian, Xuehui Cai, Tongqing An

**Affiliations:** 1 State Key Laboratory for Animal Disease Control and Prevention, Harbin Veterinary Research Institute, Chinese Academy of Agricultural Sciences, Harbin, 150069 China; 2 Heilongjiang Provincial Key Laboratory of Veterinary Immunology, Harbin, 150069 China

**Correction: Veterinary Research (2024) 55:142** 10.1186/s13567-024-01401-y

Following publication of the original article [1], the authors identified an error in Figure 2A and Figure 3A.

Regarding the originally published Figure 2A, an inaccuracy was identified in the phylogenetic tree labeling. The basal branches were initially labeled as “L1A.” While these branches do include reference strains belonging to the L1A lineage, their topological placement within the phylogenetic tree shows significant divergence from the majority of L1A reference strains. Therefore, to more accurately represent their phylogenetic position, we propose relabeling these specific branches as “L1A-unclade”.

Concerning Figure 3A, the figure legend correctly stated that the scale bar of histological lesions pictures represents 200 μm. However, due to blurriness in the original image, the scale bar was difficult to distinguish. Thus, it was necessary to replace the image with an updated version in which the scale bar is highlighted.

The original article has been corrected.
