# Supplementary material for: Recombinant characterization and pathogenicity of a novel L1C RFLP-1-4-4 variant of porcine reproductive and respiratory syndrome virus in China
Source: Vet Res. 2024 Nov 6;55:142. doi: 10.1186/s13567-024-01401-y (PMC11539553; doi:10.1186/s13567-024-01401-y)
Supplement: Supplementary file 1 — Additional file 1. Primer/probe sequence information for RT-PCR or RT-qPCR assays. [file 13567_2024_1401_MOESM1_ESM.doc]

**Additional file 1. Primer/probe sequence information for RT-PCR or RT-qPCR assay.**

| Primer/probe name | Sequence (5’-3’) | Reference |
| --- | --- | --- |
| L1-A-F | GCACTGCTTTACGGTCTCTCC |  |
| L1-A-R | GTCCAGTGCTCGCCTTC |  |
| L1-B-F | GGGAATTGTGGTTGGC |  |
| HU21-B-R | CGTCTTCAGTGGCAAC | This study |
| HU21-E-F | GAGTGAATTCTTCGCTGG |  |
| HU21-E-R | CAGTACCCACACACCCTTG |  |
| JL580-C-F | ATGCTAACCTGGCGCAAC |  |
| JL580-C-R | GGCCATCGAACAAGCAAC |  |
| JL580-D-F | GACCCCGACTGCAGGATT |  |
| JL580-D-R | TCCTTCCAGTTCGGGTTT |  |
| JL580-G-F | GGCTGCAATACTCATGGAC | [18] |
| JL580-G-R | AAACGCTTCATTGTAATCCTC |  |
| JL580-H-F | CTCAGGTCAGGTGTTTGC |  |
| JL580-H-R | GGAGCTGCTGTTGCTGTTG |  |
| JL580-I-F | TTGCCTTTTTTGTGGTGTATC |  |
| JL580-I-R | AATTTCGGCCGCATGGTTCTC |  |
| PRRSV2-UF | TTGTGCTTGCTAGGCCGC |  |
| PRRSV2-UR | ACGACAAATGCGTGGTTATCA | [23] |
| PRRSV-probe | FAM-TCTGGCCCCTGCCCA-MGB |  |
