# Supplementary material for: Recombinant characterization and pathogenicity of a novel L1C RFLP-1-4-4 variant of porcine reproductive and respiratory syndrome virus in China
Source: Vet Res. 2024 Nov 6;55:142. doi: 10.1186/s13567-024-01401-y (PMC11539553; doi:10.1186/s13567-024-01401-y)
Supplement: Supplementary file 2 — Additional file 2. Primers used for detecting cytokines by RT-qPCR assay. [file 13567_2024_1401_MOESM2_ESM.doc]

**Additional file 2. Primers used for detecting cytokines by RT-qPCR assay.**

| Gene | Primer sequence (5’-3’) | Product length (bp) |
| --- | --- | --- |
| IL-1β | TCTGCCCTGTATCCCAACTG | 144 |
|  | GACCTCTGCGTATGGCTTTCCT |  |
| IL-6 | TGGATAAGCTGCAGTCACAG | 109 |
|  | ATTATCCGAATGGCCCTCAG |  |
| IL-10 | TGAAGAGTGCCTTTAGCAAG | 138 |
|  | TCTTTGGTTTCCCTAGAATG |  |
| TNF-α | CGCATCGCCGTCTCCTACCA | 203 |
|  | TGCCCAGATTCAGCAAAGTCCAG |  |
| CCL8 | CAAGAATCACCAACAGCCAGTG | 162 |
|  | GTCCAGGTAGGAAGGTTCAAGG |  |
| MCP-1 | TACAGAAGAGTCACCAGCAG | 112 |
|  | TTATGGAGTCCTGGACCCAC |  |
| IFN-γ | GCAAGTACCTCAGATGTACC | 207 |
|  | GCTACCATTTAGGAACCTCT |  |
| IL-8 | CATAAATACGCATTCCAC | 157 |
|  | AACCTTCTGCACCCACTT |  |
| GAPDH | TGAAGGTCGGAGTGAACG | 150 |
|  | CGTGGGTGGAATCATACTGG |  |
