# Supplementary figures and images for: Recombinant characterization and pathogenicity of a novel L1C RFLP-1-4-4 variant of porcine reproductive and respiratory syndrome virus in China
Source: Vet Res. 2024 Nov 6;55:142. doi: 10.1186/s13567-024-01401-y (PMC11539553; doi:10.1186/s13567-024-01401-y)

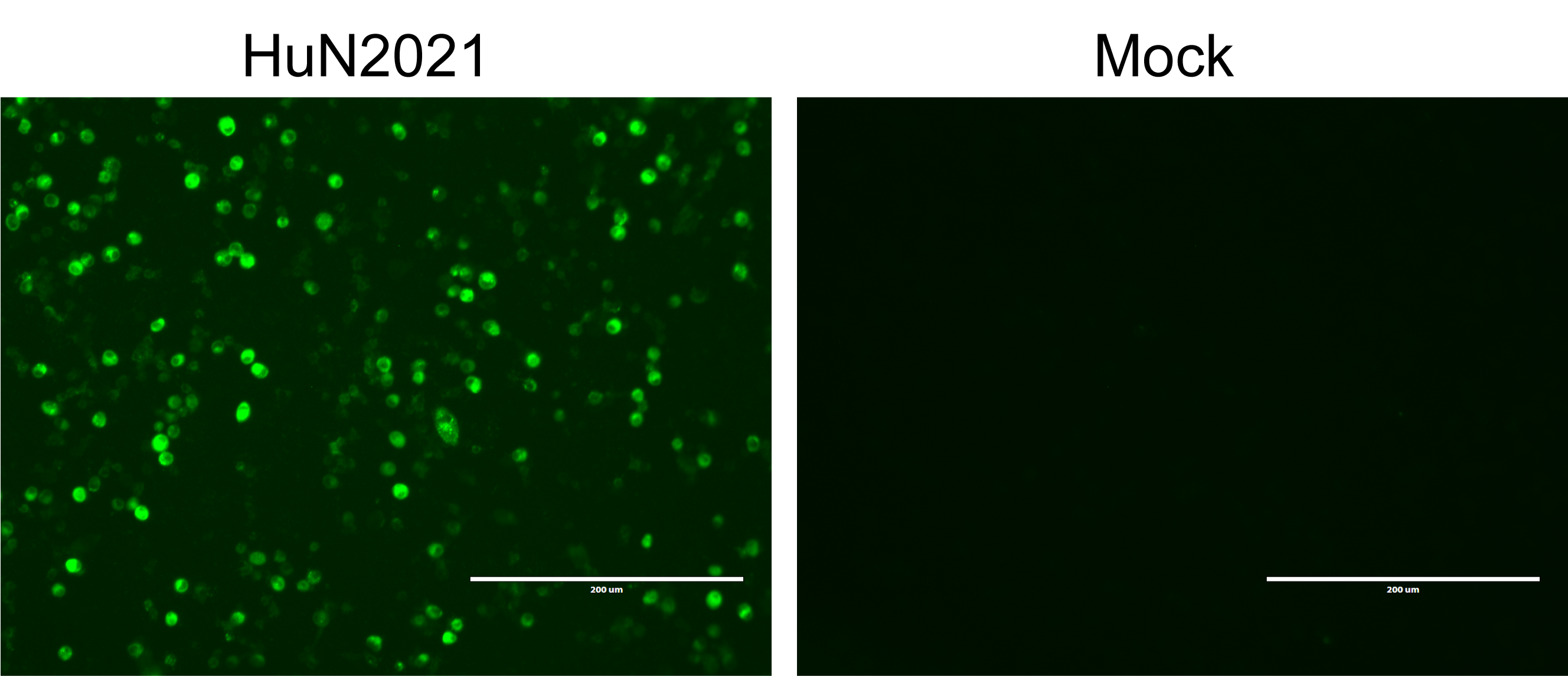

Supplement: Supplementary file 3 — Additional file 3. Isolation of the PRRSV HuN2021 strain. An indirect immunofluorescence assay was performed with an anti-PRRSV M monoclonal antibody. The bar represents 200 μm. [file 13567_2024_1401_MOESM3_ESM.tif]

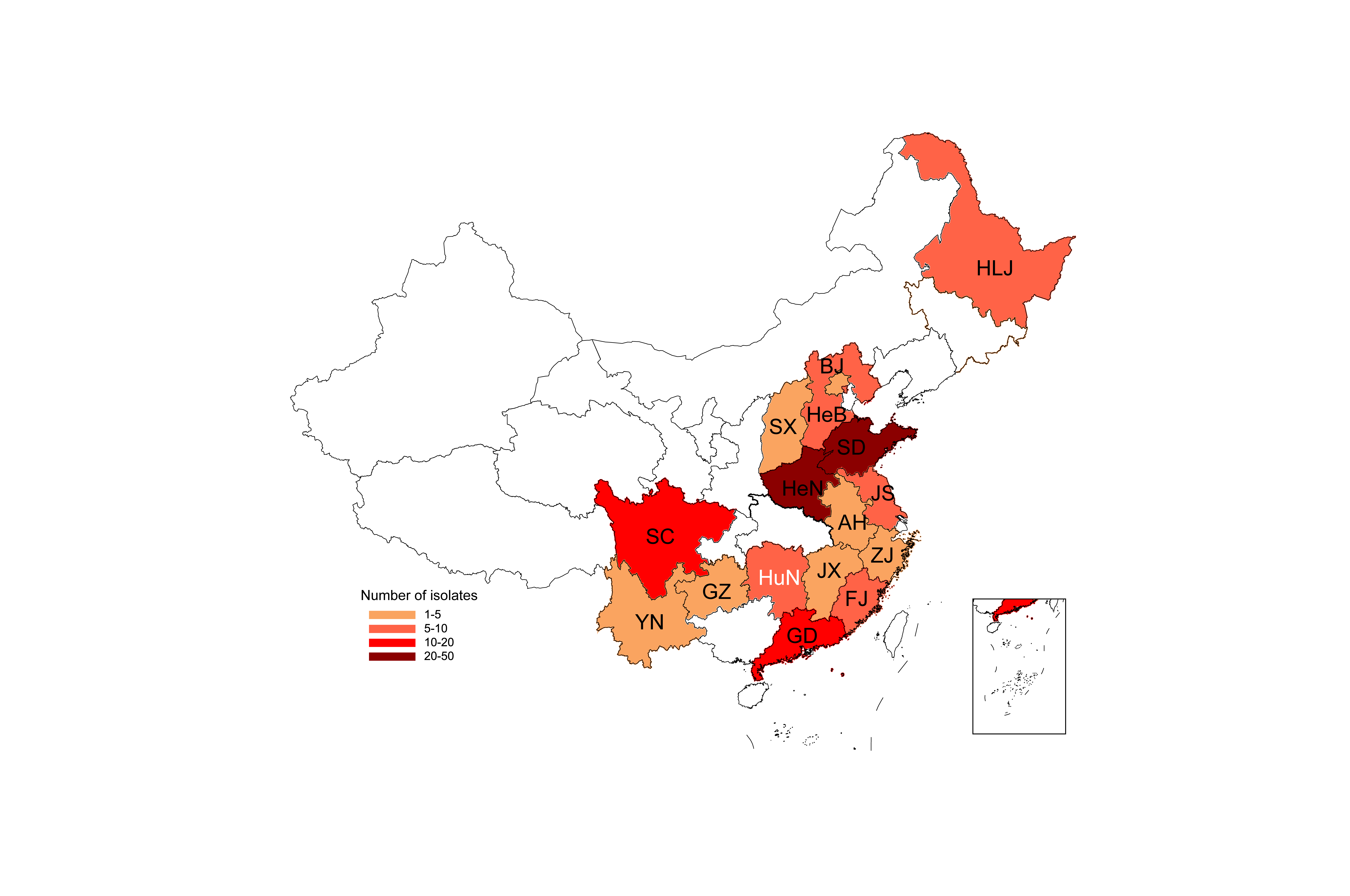

Supplement: Supplementary file 6 — Additional file 6. Geographical distribution of RFLP 1-4-4 L1C PRRSV strains isolated in China from 2016–2021. The number of RFLP 1-4-4 L1C PRRSVs in different provinces: Shandong, Henan, Guangdong, Sichuan, Hebei, Fujian, Jiangsu, Heilongjiang, Hunan, Beijing, Guizhou, Jiangxi, Shanxi, Anhui, Yunnan, and Zhejiang. [file 13567_2024_1401_MOESM6_ESM.tif]
