# Supplementary material for: Recombinant characterization and pathogenicity of a novel L1C RFLP-1-4-4 variant of porcine reproductive and respiratory syndrome virus in China
Source: Vet Res. 2024 Nov 6;55:142. doi: 10.1186/s13567-024-01401-y (PMC11539553; doi:10.1186/s13567-024-01401-y)
Supplement: Supplementary file 4 — Additional file 4. Nucleotide identity of HuN2021 with representative strains. [file 13567_2024_1401_MOESM4_ESM.doc]

**Additional file 4. Nucleotide identity of HuN2021 with representative strains.**

|  | Nucleotide identity, % | | | | | | |
| --- | --- | --- | --- | --- | --- | --- | --- |
|  | VR-2332 | QYYZ | CH-1a | JXA1 | NADC30 | NADC34 | RFLP-144 L1C variant (L1C.5) |
| NSP1α | 90.7 | 91.9 | 94.9 | 97 | 88.8 | 85.2 | 83.9 |
| NSP1β | 86 | 84.5 | 91.6 | 97.7 | 79.9 | 80.7 | 80.4 |
| NSP2 | 79.9 | 76.3 | 78.9 | 80.3 | 85.7 | 77.7 | 78.8 |
| NSP3 | 85.2 | 80.3 | 84.6 | 84.9 | 87.7 | 85.4 | 87 |
| NSP4 | 87.4 | 83.5 | 93.1 | 96.2 | 83 | 85.1 | 84.2 |
| NSP5 | 86.1 | 81 | 90.4 | 92.9 | 84.7 | 81.6 | 83.7 |
| NSP6 | 91.7 | 93.8 | 93.8 | 95.8 | 93.8 | 91.7 | 93.8 |
| NSP7 | 87.5 | 90.2 | 92.5 | 94.9 | 80.7 | 81.7 | 80.3 |
| NSP8 | 92.6 | 91.1 | 95.6 | 94.8 | 86.7 | 88.9 | 86.7 |
| NSP9 | 89.7 | 88.6 | 91.1 | 92.7 | 89.3 | 88 | 87.3 |
| NSP10 | 84.4 | 84.4 | 84.7 | 83.7 | 91.6 | 89.2 | 89.5 |
| NSP11 | 87.1 | 85.5 | 90 | 88.2 | 91 | 86.1 | 84 |
| NSP12 | 89.3 | 86.9 | 89.8 | 89.1 | 92.6 | 85.2 | 85.2 |
| GP2 | 86.4 | 84.8 | 84.8 | 84.6 | 92.2 | 83.3 | 82.6 |
| E | 88.7 | 90.5 | 86.9 | 87.4 | 93.2 | 85.6 | 86 |
| GP3 | 83.1 | 80.7 | 82.1 | 82.6 | 92.2 | 86.8 | 86.3 |
| GP4 | 87 | 85.1 | 85.5 | 85.1 | 94.2 | 95.3 | 92.4 |
| GP5 | 84.6 | 84.9 | 86.1 | 84.1 | 91.8 | 87.6 | 91.7 |
| GP5a | 84.3 | 85 | 87.9 | 85.7 | 93.4 | 91.4 | 91.4 |
| M | 89 | 88.2 | 86.9 | 87.8 | 95 | 92.2 | 94.5 |
| N | 93.3 | 88.4 | 91.7 | 91.1 | 95.7 | 93.8 | 91.9 |
| Complete genome | 85.7 | 83.7 | 86.6 | 87.6 | 88.4 | 84.7 | 84.8 |
