# Supplementary material for: Recombinant characterization and pathogenicity of a novel L1C RFLP-1-4-4 variant of porcine reproductive and respiratory syndrome virus in China
Source: Vet Res. 2024 Nov 6;55:142. doi: 10.1186/s13567-024-01401-y (PMC11539553; doi:10.1186/s13567-024-01401-y)
Supplement: Supplementary file 5 — Additional file 5. Amino acid identity of HuN2021 with representative strains. [file 13567_2024_1401_MOESM5_ESM.doc]

**Additional file 5. Amino acid identity of HuN2021 with representative strains.**

|  | Amino acid identity, % | | | | | | |
| --- | --- | --- | --- | --- | --- | --- | --- |
|  | VR-2332 | QYYZ | CH-1a | JXA1 | NADC30 | NADC34 | RFLP-144 LIC variant (L1C.5) |
| NSP1α | 94.3 | 96.6 | 95.5 | 96.6 | 95.5 | 94.9 | 93.2 |
| NSP1β | 83.2 | 81.2 | 86.1 | 96 | 74.8 | 76.2 | 76.7 |
| NSP2 | 74.8 | 71.1 | 73.4 | 75.9 | 84.5 | 73.4 | 73.2 |
| NSP3 | 92.2 | 86.5 | 90 | 90 | 92.6 | 90.4 | 93.9 |
| NSP4 | 93.1 | 91.2 | 95.1 | 98.5 | 92.2 | 93.6 | 92.2 |
| NSP5 | 89.4 | 87 | 91.2 | 92.4 | 90 | 87.1 | 90 |
| NSP6 | 93.8 | 100 | 100 | 100 | 100 | 87.5 | 93.8 |
| NSP7 | 88.8 | 92.3 | 94.2 | 96.1 | 83.8 | 86.5 | 84.6 |
| NSP8 | 93.3 | 93.3 | 93.3 | 93.3 | 91.1 | 91.1 | 88.9 |
| NSP9 | 96.9 | 95.9 | 97.2 | 97.5 | 96.4 | 96.4 | 96.6 |
| NSP10 | 94.6 | 93.7 | 93.4 | 93.4 | 96.6 | 96.4 | 96.1 |
| NSP11 | 93.7 | 94.2 | 96.4 | 96.9 | 97.3 | 95.1 | 94.2 |
| NSP12 | 94.1 | 95.4 | 94.8 | 96.1 | 96.7 | 91.5 | 92.8 |
| GP2 | 86.4 | 86.4 | 84.8 | 83.3 | 88.7 | 81.3 | 82.5 |
| E | 85.1 | 86.5 | 83.8 | 86.5 | 94.6 | 87.8 | 87.8 |
| GP3 | 79.6 | 79.2 | 78 | 78.8 | 90.2 | 85.5 | 84.7 |
| GP4 | 87.2 | 85.5 | 87.2 | 87.2 | 96.1 | 97.2 | 92.2 |
| GP5 | 82.6 | 82.1 | 84.6 | 83.6 | 94.5 | 91 | 94.5 |
| GP5a | 84.3 | 85 | 87.9 | 85.7 | 93.4 | 91.4 | 91.4 |
| M | 93.1 | 92 | 92.6 | 93.7 | 98.9 | 94.3 | 98.3 |
| N | 93.5 | 93.5 | 91.9 | 91.9 | 96 | 93.5 | 93.5 |
